# Quality-of-Service-Centric Design and Analysis of Unmanned Aerial Vehicles

**DOI:** 10.3390/s22155477

**Published:** 2022-07-22

**Authors:** Sudhanshu Kumar Jha, Shiv Prakash, Rajkumar Singh Rathore, Mufti Mahmud, Omprakash Kaiwartya, Jaime Lloret

**Affiliations:** 1 Department of Electronics and Communication, University of Allahabad, Prayagraj 211002, Uttar Pradesh, India; skjha@allduniv.ac.in (S.K.J.); shivprakash@allduniv.ac.in (S.P.); 2 Department of Computer Science, Cardiff School of Technologies, Cardiff Metropolitan University, Cardiff Llandaff Campus, Cardiff CF5 2YB, UK; rsrathore@cardiffmet.ac.uk; 3 Department of Computer Science, Nottingham Trent University, Clifton Campus, Nottingham NG11 8NS, UK; mufti.mahmud@ntu.ac.uk; 4 Computing and Informatics Research Centre, Nottingham Trent University, Nottingham NG11 8NS, UK; 5 Medical Technologies Innovation Facility, Nottingham Trent University, Nottingham NG11 8NS, UK; 6 Department of Communications, Universitat Politècnica de València, 46022 Valencia, Spain; jlloret@dcom.upv.es

**Keywords:** unmanned aerial vehicles, quality of service, UAV design, blended wing body

## Abstract

Recent years have witnessed rapid development and great indignation burgeoning in the unmanned aerial vehicles (UAV) field. This growth of UAV-related research contributes to several challenges, including inter-communication from vehicle to vehicle, transportation coverage, network information gathering, network interworking effectiveness, etc. Due to ease of usage, UAVs have found novel applications in various areas such as agriculture, defence, security, medicine, and observation for traffic-monitoring applications. This paper presents an innovative drone system by designing and developing a blended-wing-body (BWB)-based configuration for next-generation drone use cases. The proposed method has several benefits, including a very low interference drag, evenly distributed load inside the body, and less radar signature compared to the state-of-the-art configurations. During the entire procedure, a standard design approach was followed to optimise the BWB framework for next-generation use cases by considering the typically associated parameters such as vertical take-off and landing and drag and stability of the BWB. Extensive simulation experiments were performed to carry out a performance analysis of the proposed model in a software-based environment. To further confirm that the model design of the BWB-UAV is fit to execute the targeted missions, the real-time working environments were tested through advanced numerical simulation and focused on avoiding cost and unwanted wastages. To enhance the trustworthiness of this said computational fluid dynamics (CFD) analysis, grid convergence test-based validation was also conducted. Two different grid convergence tests were conducted on the induced velocity of the Version I UAV and equivalent stress of the Version II UAV. Finite element analysis-based computations were involved in estimating structural outcomes. Finally, the mesh quality was obtained as 0.984 out of 1. The proposed model is very cost-effective for performing a different kind of manoeuvring activities with the help of its unique design at reasonable mobility speed and hence can be modelled for high-speed-based complex next-generation use cases.

## 1. Introduction

Unmanned aerial vehicles’ (UAV) design and development, focusing on next-generation use cases, is one of the growing research themes for smart city environments [1,2]. The smart-service-centric UAV domain includes aerial delivery, smart healthcare, smart home, Internet of vehicles, design simulation, pollution monitoring, smart agriculture, disaster management, industrial Internet of things, and green mobility [3,4]. It has emerged as a prominent development area to revolutionise UAV-assisted next-generation use cases in day-to-day life [5]. A fixed-wing UAV is a type of unmanned aerial vehicle. In plain sight, it looks like a scaled-down model of a passenger aircraft with propellers for its thrust and sometimes for achieving vertical take-off and landing [6]. A fixed-wing UAV is controlled either remotely by a human operator or autonomously via an onboard computational mechanism [7]. A fixed-wing UAV can fly by utilising the lift generated by the aircraft’s forward motion and the shape of its wings. Self-propelled fixed-wing UAVs typically rely on the thrust generated by a propeller turned by an internal combustion engine or electric motor. Gliders are launched either by a winch launcher or by being towed by another aircraft. In both cases, the ailerons, elevator, and rudder control the drone’s roll, pitch, and yaw [8]. In addition to batteries and conventional petrol or diesel-based engines, a powered fixed-wing UAV can operate on other energy sources such as solar power [9]. The major classifications of UAVs are conventional fixed-wing UAVs, canard configuration, flying wing configuration, and blended-wing-body configuration.

A conventional fixed-wing UAV is a scaled-down model of a passenger aircraft with a tractor or pusher-type propeller to provide the necessary thrust for its forward motion [10]. The vehicle contains a fuselage body with size as required by the payload, a propeller for thrust, a rectangular wing for the necessary lift production, and a conventional tail to achieve pitching and yawing moments [11]. The canard-wing UAV configuration is also a scaled-down model of a canard aircraft where a small forewing is placed forward of the main wing of a fixed-wing aircraft. These are considered because of their flight control, instability, trim, and modified flow over the main wing parameters [12]. These are not highly used in the UAV category, as the disadvantages outweigh the advantages. Simple configurations are available that are easy to manufacture and more suited for the required applications [13]. The flying wing configuration is the configuration with no definite fuselage. It has no horizontal tail surfaces, resulting in lesser drag and a larger wing area with a better range and endurance than the conventional UAV configuration. The construction of this UAV is simple in the sense that the required sections of the air foils are made along with spars, and skin is attached over the sections [14].

**Figure 1:**
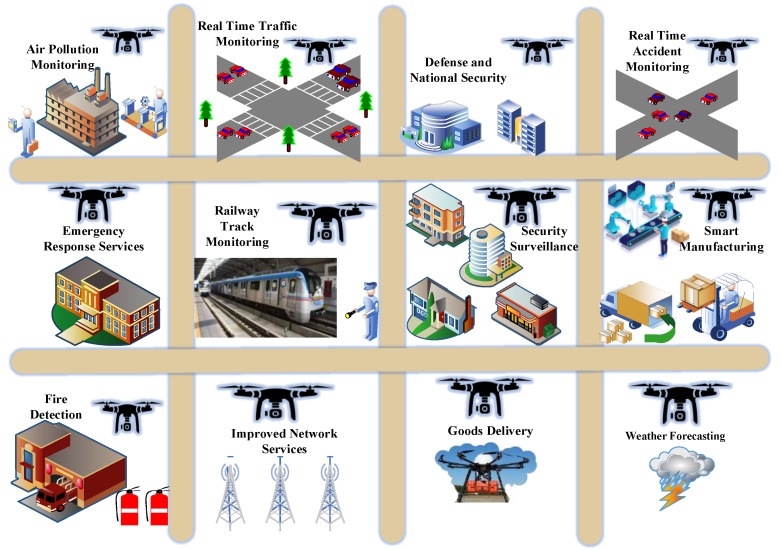
Next-generation use cases for drones.

The blended wing body (BWB) is a tailless design that integrates the wing and the fuselage. It consists of a middle section and an outer section wing, whereas the part in between is the blending area where the central body smoothly connects and blends into the wing geometry. It facilitates several advantages such as a low wetted area to internal volume ratio, the potential for elliptic lift distribution, smooth varying cross-section distribution, and adequate engine installation space and enables several next-generation use cases for drones, as shown in Figure 1. In this context, the objective of this research work is to efficiently design a BWB-based UAV to execute the targeted missions for the high-speed-centric complex next-generation UAV use cases. The proposed method has novel performance benefits, including a very low interference drag, evenly distributed load inside the body, and less radar signature than the other configurations. These performance benefits have been confirmed with the help of extensive simulation experiments, which were performed to carry out a performance analysis of the proposed model in a software-based environment. To enhance the trustworthiness, computational fluid dynamics (CFD) analysis and grid convergence test-based validation were also conducted. Two different grid convergence tests were conducted on the induced velocity of the Version I UAV and equivalent stress of the Version II UAV. Further, Table 1 reflects the list of abbreviations for better understanding the different terms used in this paper. The contributions of this research article can be outlined in a point-wise fashion as follows:

1. This paper presents a noble, innovative idea for designing and developing a blended-wing-body (BWB)-based configuration system for UAVs, especially for next-generation high-speed drone use cases.
2. The proposed new design has innumerable expediency, such as very low interference drag, evenly distributed load inside the body, and less of a radar signature for security.
3. Blended-wing-body (BWB) framework has been optimised for next-generation use cases considering typical parameters such as vertical take-off and landing, drag, and stability of the blended wing body.
4. The experimental result analysis of the BWB-based UAV attests the performance benefits in terms of cost-effectiveness in performing different kinds of manoeuvring activities due to its unique design at reasonable mobility speed and hence can be applied for the high-speed-based complex next-generation UAV use cases.

Further, the paper is structured as follows. Section 2 discusses the literature review. Section 3 presents the details of the proposed model. Section 4 elaborates on the implementation environments and analysis of results, followed by a conclusion with future scope in Section 5.

## 2. Related Work

### 2.1. UAV Design Considering Networking

Mobility-based networking in the sky was initially introduced considering commercial UAVs as routers [15]. It enables in-flight Internet by addressing two major issues: resource management and Internet over satellite support. Data access architecture, clustering of aerial nodes focusing on stability, and a reliable data dissemination scheme were the major developments of the work. However, it has a novel application for ad hoc networking scenarios in aerial environments. Towards highlighting the significance of ad hoc networks in civilian applications, a greedy perimeter stateless routing (GPSR) was suggested in [16]. This paper discusses the three conventional types of UAV configurations by comparing the parameters such as ease of manufacturing process, stability, ease of hand launch, mass, and ground clearance and allotting the weightage between one and five. The conventional configuration with a high-wing UAV is efficient for basic applications [17].

The UAV networking-centric use case design challenges have been reviewed critically with possible solutions [18]. Various prototype designs have been presented as a possible solution for cyber physical system applications. Similarly, as a use case of a UAV networking system, a UAV swam architecture has been suggested focusing on network cooperation among UAVs [19]. Another use case of connected drones has been investigated focusing on parcel delivery [20]. These UAV networking-centric use cases have potential for deployment as a business-case scenario. However, these studies are very general without much focus on quality of service in the targeted UAV use case.

### 2.2. UAV Design Considering Airfoils

In the category of the conceptual design of a blended-wing-body MALE UAV, this paper discusses the conceptual design of a medium-range long-endurance UAV in a blended-wing-body configuration. The airfoils, namely MH-108, MH-18, and FX 76-MP-120, were shortlisted for the design, and three different modifications were designed using CAD software and performance analysis carried out using CFD. Stability analysis was also carried out, concluding that the configuration with a vertical tail in the wingtips gives better results in both CFD and stability analyses [21]. Mosquera et al. [22] discussed the performance optimisation of fixed-wing unmanned aerial vehicles and a wind tunnel testing of a blended-wing-body UAV. They tested the stability parameters and found it to have good stability conditions, except for its take-off and landing sections.

Panagiotou et al. [23] presented three different designs of a passenger aircraft in the blended-wing-body configuration. The key characteristics of their design were flight and field performance, static stability, balance, and weight. They analysed the passenger accommodation. However, the comparisons among them were not presented. Moreover, Panagiotou et al. [24] further presented the aerodynamic study of four different blended-wing-body platforms. Design parameters such as wing sweep angle, number of airfoils, and the aspect ratio have been studied across the four models with the help of CFD software. Three different airfoils used for the blended wing body were MH-91, E374, and NLF1015 from the fuselage centreline to the wingtip. The Reynolds-Averaged Navier–Stroke (RANS) equations coupled with the low Reynolds turbulence model were used to obtain accurate shear and average stress on the wing surfaces. Turbulence models such as k-omega SST and Spalart–Allmaras were used for the external flow analysis.

### 2.3. UAV Design Considering Wing Geometry

Parisa et al. [25] studied the conceptual design of a VTOL-integrated blended-wing-body UAV. In this layout, a propeller in the duct located in the middle of the fuselage section is used for achieving vertical take-off and landing. Two modes of the same model were tested experimentally, and the thrust from the four propellers submerged in the fuselage was calculated at a different angle of attack. This range and endurance are limited by the capacity of the battery used. Further, Salazar-Jiménez et al. [26] discussed a deep analysis of two main aspects: wing geometry and aerodynamic behaviour. They utilised the CFD tools to estimate the various parameters with precision. Next, Rajesh A et al. [27] implemented a particular winglet for the UCAV wing. They further concluded that the efficiency is much improved due to the reduction in drag and increment in lift-to-drag ratio. Moreover, Escobar-Ruiz et al. [28] have used solar cells specifically as the main power source in designing unmanned aerial vehicles (UAV). There are three basic building blocks in this mechanism: preliminary design, conceptual design, and computational fluid dynamics analysis.

Furthermore, Lian et al. [29] have found out that there exists a direct coupling between the efficiency of propulsion and aerodynamics. They have developed the integrated analysis method for measuring the performance of different configurations having various engine positions. Additionally, Chiesa et al. [30] have used the proprietary tools for the preliminary and conceptual design of UAVs. They have significantly contributed in terms of modern configurations for MALE UAVs. In addition, Grendysa et al. [31] have successfully discovered the optimal geometric parameters. They have also developed the optimising method for light UAVs. Aleisa et al. [32] investigated generic UCAVs by considering two unique characteristics: low-speed aerodynamics and flow field. Finally, Arnhem et al. [33] have presented the experimental and numerical studies. For aircraft configuration, aerodynamic interaction has been carried out. There are several next-generation UAV use-case scenarios, including applications in the Internet of things [34], and vehicular environments [35] have been investigated.

## 3. Proposed Quality-of-Service Provisioning in UAV-Assisted Networks

**Figure 2:**
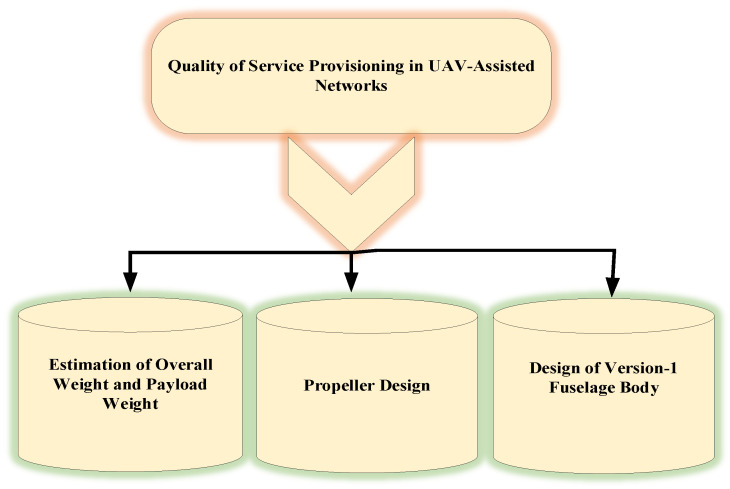
Main building blocks in quality-of-service provisioning in UAV-assisted networks.

This section presents the proposed quality-of-service provisioning framework for UAV-assisted networks (QSPU) in detail. In particular, a network model comprising a mobility model for UAV-assisted ad hoc networking is presented. The service parameters are derived, including UAV connectivity, route-oriented service lifetime, and service delay. A route selection approach based on the service parameters and broadcasting optimisation technique has been developed for smart service delivery. Figure 2 illustrates the main building blocks in quality-of-service provisioning in UAV-assisted networks. Detailed modelling is presented in following subsections using the symbols in Table 2. It is clarified that the proposed design of a BWB-based UAV is suitable for next-generation high-speed drone use cases including surveillance considering the quality-of-service-centric modelling of drones. The BWB-centric airframe has potential features for these use cases such as efficient lift and minimal drag considering the wide airfoil along with high-lift wings for better speed control while in operation. It has low fuel consumption as well as a large payload area. The BWB airframe has minimum skin drag due to the reduction in total wetted area. The wing root area is much thicker as compared to conventional design; thereby, optimal weight reduction can be witnessed.

### 3.1. Estimation of Overall Weight and Payload Weight

Payload is chosen as a stabilised four-sensor UAV gimbal, wherein the weight of the payload is 1.77 kg, has 360-degree continuous pan rotation, the HD video output is 720 p, and it has an adopted moving target indicator. The final payload weight is attained by adding all the accessory components as 4.5 kg. For this work, the payload weight is assumed to be 5 kg. The payload weight plays a predominant role in the estimation of the overall weight of the UAV with the help of historical relationships. Thus, the fine-tuned fieldwork was executed on the estimation of payload weight for the shortlisted missions. The estimated payload weight is 5 kg, expressed in Equation (1). From the historical relationship,

(1) $$\begin{array}{l} W_{\mathrm{Pl}}=0.298096989{\;W}_{O}\;\\ 5=0.298096989{\;W}_{O}\;\Rightarrow W_{O}=\frac{5}{0.298096989}\;\Rightarrow W_{O}=16.7731\;\mathrm{kg} \end{array}$$

### 3.2. Propeller Design

The propeller is designed based on the calculations involving total weight and rate of climb. The propeller’s pitch and diameter were calculated, and the CAD model was generated. A design standard National Advisory Committee for Aeronautics (NACA 25112) airfoil profile is used for the conceptual design of the propellers [36]. It is highlighted that NACA is an appropriate design standard for UAVs. It is a series of thoroughly tested airfoils and a numerical designation for each airfoil. The five-digit number represents the airfoil section’s critical geometric properties. The NACA 25112 design standard is very efficient since it has low drag and increased lift forces at relatively low wind speeds for UAVs. Since we are incorporating a co-axial propeller system, both clockwise propellers and the anti-clockwise propeller are designed using CAD design software. The CAD models of the horizontal take-off and landing (HTOL) and vertical take-off and landing (VTOL) propellers are shown in result analysis, Section 4.2.

(2) $$T=0.5*\rho *A*\left[{\left(V_{e}\right)}^{2}-{\left(V_{o}\right)}^{2}\right]=\frac{100{\;\%\;\mathrm{of}\;\mathrm{the}\;\mathrm{UAV}}^{’}s\;\mathrm{weight}\;}{2}$$

(3) $$\theta =\;\mathrm{arctangent}\;\left(\frac{P}{2*\pi *r}\right)$$

(4) $$b=\frac{8*\pi *\left(\frac{\sin\left(\theta \right)*\left(\tan\left(\theta \right)-\frac{1}{1.2}*\tan\left(\theta \right)\right)}{\left(1+\frac{1}{1.2}*\tan\left(\theta \right)\right)}\right)*r}{{n*C}_{L}}\;$$

(5) $$C_{L}=\frac{2*L}{{\rho V}^{2\;}A}$$

### 3.3. Design of Version I Fuselage Body

The first iteration of the design was based entirely on the literature survey, and the relationship from the literature calculated the chord lengths at different sections. For the first iteration, a single airfoil MH-91 is used for different sections and designed in CAD software. It is highlighted that we have used a unique combination of single Martin Hepperle airfoil (MH-91) because longitudinal stability can always be achieved by selecting a suitable combination of sweep and twist [37]. For best all-round performance, airfoils with low moment coefficients are better suited, although MH-91s need smaller amounts of twist, which results in a broader speed range without paying too many penalties off the design point. In general, the value for wing loading is more than 100 kg/m^2^ for high-loading UAVs, the value for wing loading varies from 50 kg/m^2^ to 100 kg/cm^2^ in the case of medium-loading UAVs, and finally, the value for wing loading is less than 50 kg/m^2^ for low-loading UAVs. This work assumes the wing loading as 20 kg/m^2^.

(6) $$\begin{array}{l} S_{\mathrm{wing}}=\frac{W_{o}}{\frac{W}{S}}\;\\ S_{\mathrm{wing}}=\frac{16.7731}{20}=0.838655\;\mathrm{kg}/m^{2} \end{array}$$

The aspect ratio is assumed to be four; thus,

(7) $$\begin{array}{l} \mathrm{AR}=\frac{b_{\mathrm{wing}}{}^{2}}{S_{\mathrm{wing}}}\\ \mathrm{AR}=\frac{b_{\mathrm{wing}}{}^{2}}{0.838655\;}\Rightarrow b_{\mathrm{wing}}=\sqrt{0.838655*4}=1.832\;m \end{array}$$

In this work, the norm is to have the fuselage length be about 70–80% of the wingspan.

(8) $$L=ƞ\;*\;b_{\mathrm{wing}}$$

where ƞ is taken as 0.7, and then

(9) $$L_{\mathrm{UAV}}=0.7*1.832=1.2824\;m\;$$

The relationship between wingspan, chord length, and wing area,

(10) $$\begin{array}{l} S_{\mathrm{wing}}={b*C}_{\mathrm{wing}-\mathrm{root}}\;\\ C_{\mathrm{wing}-\mathrm{root}}=\frac{0.838655}{1.832\;}=0.458\;m \end{array}$$

From the literature survey, it is found that λ = 0.5 is more suitable to provide low drag with high lift at a positive angle of attack; therefore, in this work, λ = 0.5 is used.

(11) $$\mathrm{Taper}\;\mathrm{ratio}\;\left(\lambda \right)=\frac{C_{t}}{C_{r}}\;\Rightarrow C_{\left(span\;\%\right)}$$

In this work, the high wing configuration is planned, so to calculate the chord of any span-wise location, the b/2 is important.

For span-wise chord estimations,

(12) $$\frac{C}{C_{r}}=1-\left[2\left(1-\lambda \right)\frac{y}{b}\right]\;$$

At 25% of the span of both sides,

$$\begin{array}{l} C_{25\%}=C_{\mathrm{wing}-\mathrm{root}}\left[1-\left[2\left(1-\lambda \right)\frac{y}{b}\right]\right]\Rightarrow 0.458\;\left[1-\left[2\left(1-0.5\right)\frac{0.229}{\left[1.832\right]}\right]\right]\\ C_{25\%}=0.40075 \end{array}$$

At 50% of the span of both sides,

$$\begin{array}{l} C_{50\%}=C_{\mathrm{wing}-\mathrm{root}}\left[1-\left[2\left(1-\lambda \right)\frac{y}{b}\right]\right]\Rightarrow 0.458\;\left[1-\left[2\left(1-0.5\right)\frac{0.458}{\left[1.832\right]}\right]\right]\\ C_{50\%}=0.3435 \end{array}$$

At 50% of the span of both sides,

$$\begin{array}{l} C_{75\%}=C_{\mathrm{wing}-\mathrm{root}}\left[1-\left[2\left(1-\lambda \right)\frac{y}{b}\right]\right]\Rightarrow 0.458\;\left[1-\left[2\left(1-0.5\right)\frac{0.687}{\left[1.832\right]}\right]\right]\\ C_{75\%}=0.28625 \end{array}$$

Through these data, the main body of the BWB-UAV is modelled [38], where b—wingspan, λtaper ratio, $\mathrm{Wingspan}\;\left(b\right)=1.832\;m,$ $\frac{b}{2}=\frac{1.832\;}{2}=0.916\;m$

(13) $$\begin{array}{l} \mathrm{Sweep}\;\mathrm{Angle}=\tan^{-1}\left(\frac{C_{\mathrm{wing}-\mathrm{root}}-C_{\mathrm{Wing}-\mathrm{tip}}}{\mathrm{Half}\;\mathrm{of}\;\mathrm{the}\;\mathrm{Wingspan}}\right)\\ \mathrm{Sweep}\;\mathrm{Angle}=\tan^{-1}\left(\frac{0.458-0.229}{0.916}\right)\Rightarrow \tan^{-1}\left(0.25\right)\Rightarrow 14.04{}^{\circ} \end{array}$$

**Figure 3:**
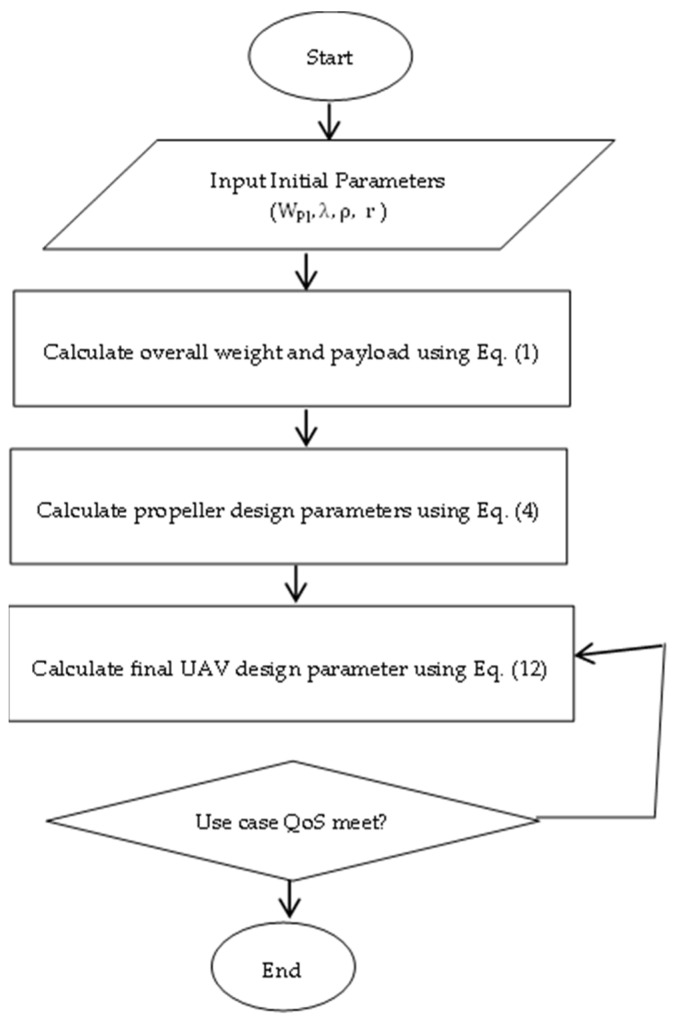
Workflow of the proposed BWB-based UAV design.

A complete workflow of the proposed UAV framework is shown in Figure 3.

## 4. Experimental Results and Discussion

### 4.1. Experimental Environment

In this section, simulation experiments were performed to carry out a performance analysis of the proposed model in a software-based environment. The BWB-UAV is constructed with the help of a standard formula and advanced modelling tool. To further confirm that the modelled design of BWB-UAV is fit to execute the targeted missions, the real-time working environments were tested through advanced numerical simulation and focused on avoiding cost and unwanted wastages. ANSYS Fluent is used to estimate the drag generation over this proposed UAV at the various working manoeuvrings. To enhance the trustworthiness of this said CFD analysis, grid convergence test-based validation is also planned. Through the help of a standard formula, the design parameters, components, boundary conditions, and their specifications are obtained. Then, fluid–structure interaction will be carried out using different materials for HTOL and VTOL operations to pick suitable materials to resist under aforesaid working environmental loads.

CFD is a flexible cum advanced tool mainly used to predict the aerodynamic forces (lift and drag) over the BWB-UAV and aerodynamic pressures acting on the BWB-UAV. In CFD, discretization is an important process, which comprises transforming continuous functions into discrete functions that provide spacing between points. The maximum velocities are assumed to be 75 m/s for forward speed and 50 m/s for VTOL operation; therefore, the flow is incompressible, so a pressure-based solver is used. Because of the complicated design, the turbulence formation is quite high, so the standard k-epsilon turbulence model is used with second-order quality. The operating pressure is picked as 101,325 Pa.

The maximum velocities were obtained as 75 m/s and 50 m/s, so the same inputs were given as initial conditions for these computations. The no-slip condition was given on the surface of the UAV, and the free slip condition was given on the outer wall. The cylindrical shape-based wall was used as a control volume with the industry-accepted dimension range (10 times the fuselage length and 15 times the wingspan). SIMPLE-based velocity and pressure coupling was used, and all the major equations were imposed with second-order quality. The maximum finalised velocity was 75 m/s, in which the flow was incompressible, and the density was constant. So, the pressure-based solver was chosen, and we can easily estimate the fluid properties in a given control volume. The main fluid parameters, such as pressure and volume, were computed at each node of a control volume. Due to the presence of the propeller, the chance of turbulence occurrences was high. So, the turbulent flow was chosen. The second order was chosen because of the high commitment to capturing the turbulence flow effectively.

### 4.2. Result Analysis

**Figure 4:**
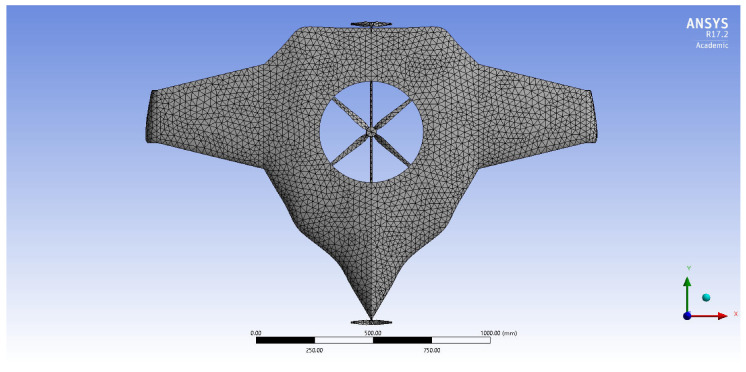
Mesh of Version II of BWB-UAV.

Mostly lightweight with high load resisting characteristics, materials have been imposed on the construction of UAV manufacturing. In this regard, various lightweight materials were imposed under these structural computations subjected to aerodynamic loading conditions. Finite element analysis-based computations were involved in estimating structural outcomes, wherein an un-structural mesh is imposed in this simulation. The fine mesh facility was used on complicated components such as vertical and horizontal propellers. The curvature and area proximity-based mesh facilities were imposed on the entire structure. Finally, the mesh quality was obtained as 0.984 out of 1. A grid convergence test was conducted, and a suitable mesh case was found, in which six different mesh cases were used. Figure 4 reflects the discretised structure of the UAV.

**Figure 5:**
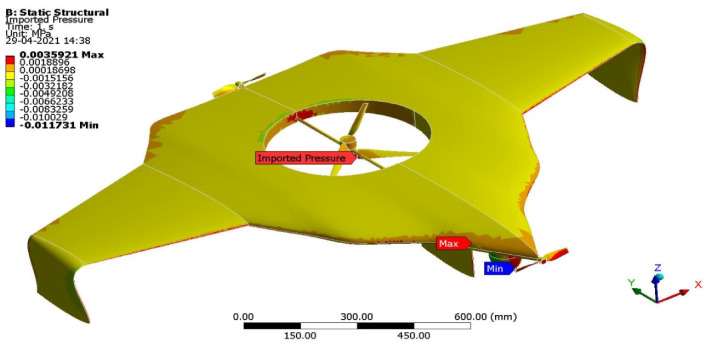
Aerodynamic loads imported for Version II.

The aerodynamic load was transferred through the FSI coupling scheme, and the UDL was applied on the surface of the UAV. The fixed support was given at all the root sections of the propellers. Major lightweight materials and their materials properties were imposed for this investigation. The complete boundary conditions are expressed in Figure 5.

**Figure 6:**
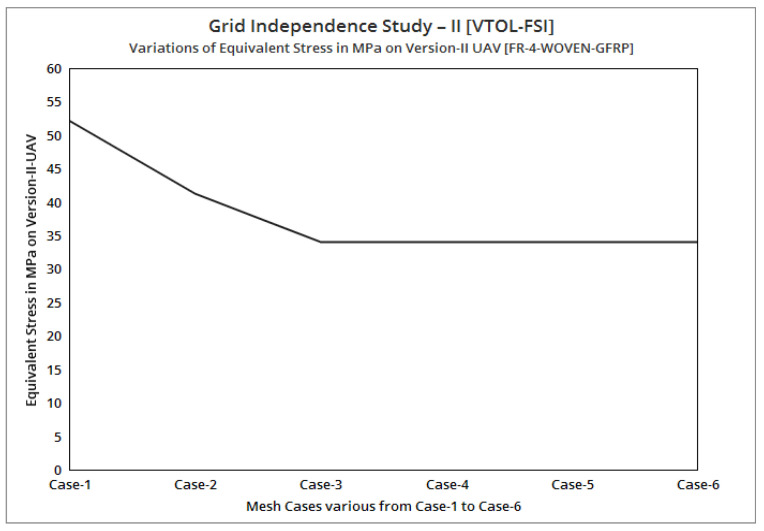
Grid independent study II (VTOL–FSI).

Figure 6 reflects the comparative analysis of grid convergences test II, wherein the grid convergence conduced on the variations of the equivalent stress while the Version II UAV moved in the vertical direction and was made-up of an FR-4-woven-GFRP-based composite. From Figure 5, mesh case three has been chosen as the best convergence due to its integrated effects.

**Figure 7:**
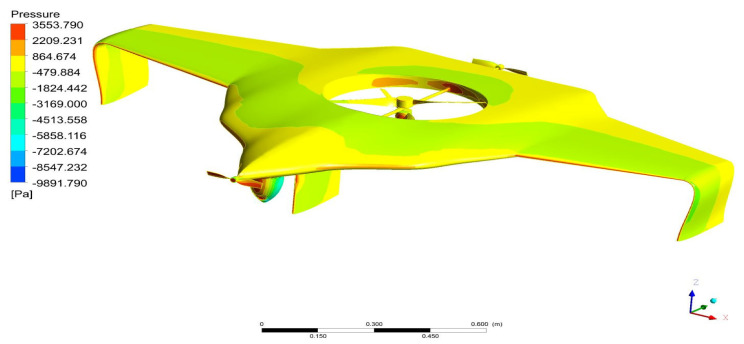
Pressure variations on the UAV under the forward speed.

**Figure 8:**
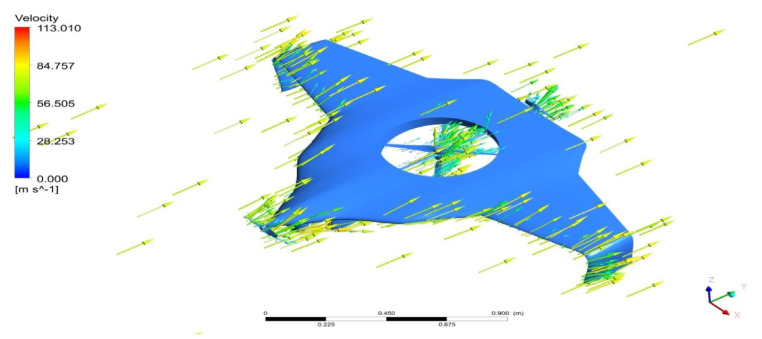
Velocity distributions over the UAV under the forward speed.

**Figure 9:**
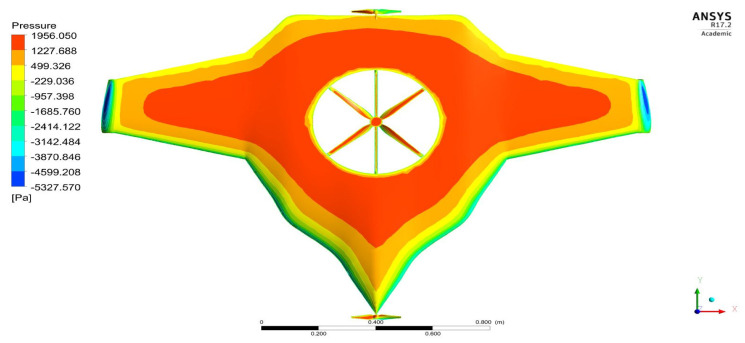
Pressure variations on UAV under VTOL.

**Figure 10:**
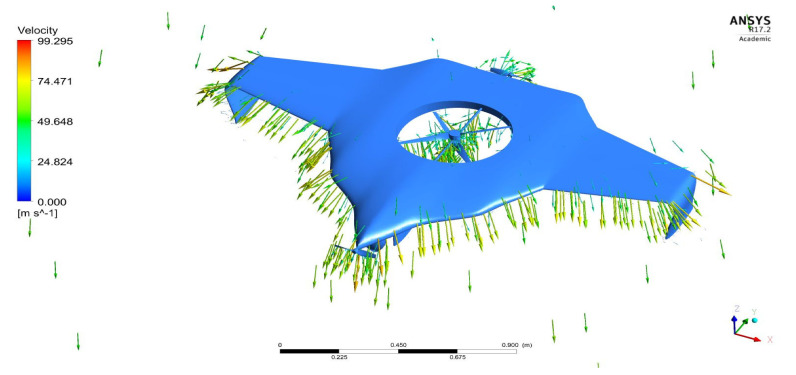
Velocity variations on UAV under VTOL.

**Figure 11:**
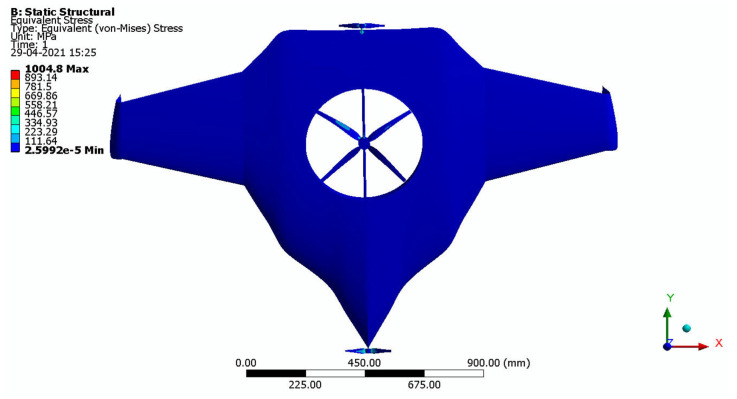
Equivalent stress.

As per the aforementioned boundary conditions, the CFD computation was executed, and the results are revealed in Figure 7, Figure 8, Figure 9, Figure 10 and Figure 11. Figure 8 and Figure 10 reveal the aerodynamic pressure variations on Version II of the BWB-UAV under forwarding speed and VTOL manoeuvring, respectively. In both the pressure variations, the positive pressure belonged to static pressure increment due to the movement of the UAV, and negative pressure belonged to dynamic pressure increment due to the movement of the UAV. Figure 8 and Figure 10 reveal the velocity distributions over Version II of the BWB-UAV under forwarding speed and VTOL manoeuvring, respectively. The aerodynamic forces such as drag, lift, and side forces were captured for both forward speed and VTOL manoeuvrings to set the working conditions of the components of Version II of the BWB-UAV. Mainly, the estimation of drag force will provide the reaction and thereby overcoming of the UAV through its propeller and corresponding rotational speeds. Additionally, the estimation of lift will provide the reaction and execution of the UAV when executing hovering through its propeller and its corresponding rotational speeds.

**Figure 12:**
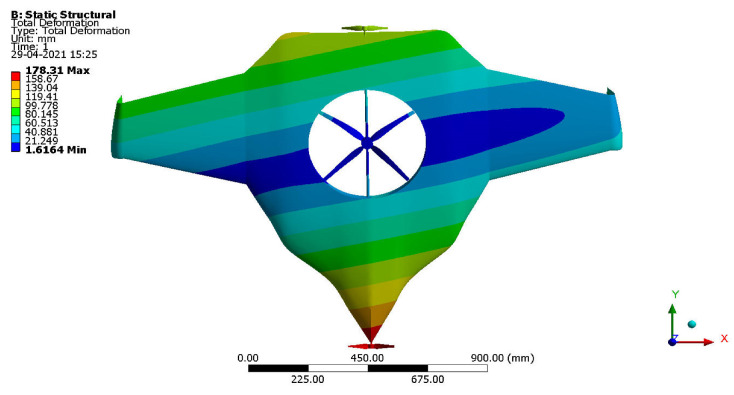
Total deformation.

**Figure 13:**
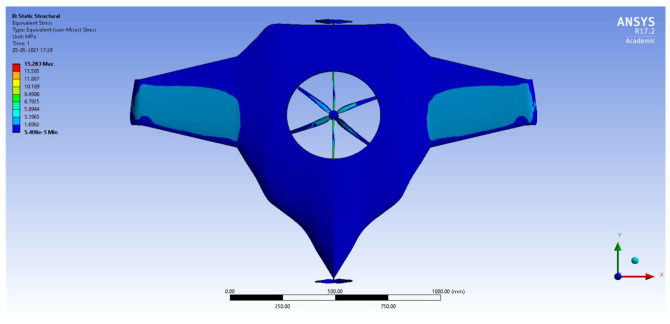
Equivalent stress.

**Figure 14:**
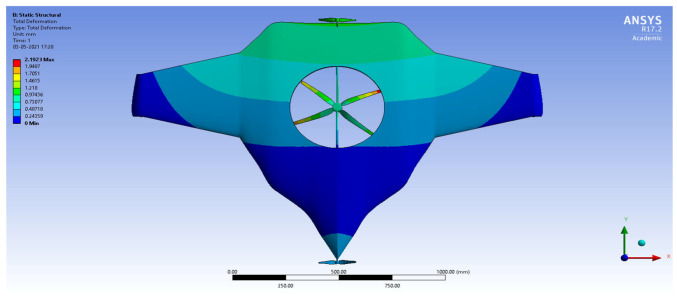
Total deformation.

**Figure 15:**
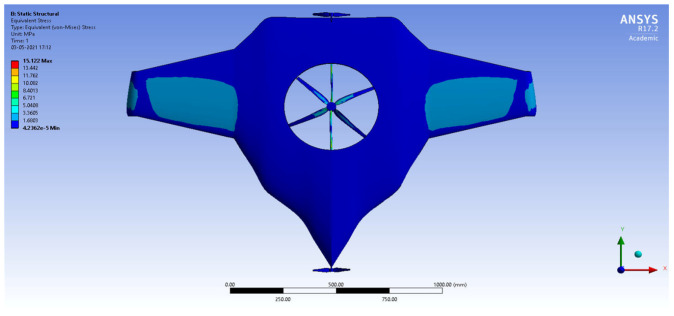
Equivalent stress.

**Figure 16:**
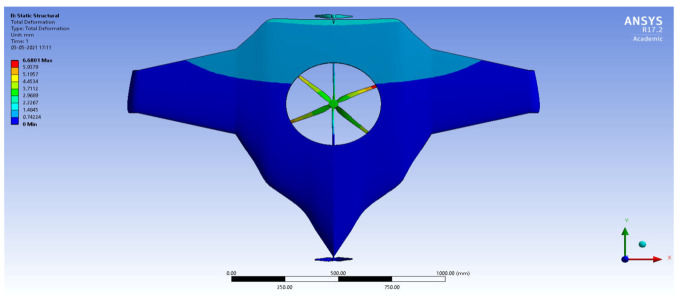
Total deformation.

After the extraction of aerodynamic simulation, the loads were transferred to the external surface of the UAV. Both forward speed and VTOL manoeuvrings were investigated under the maximum aerodynamic loads. The comprehensive computations were executed per the above boundary conditions and concluded the grid convergence outcome. The structural results are revealed in Figure 11, Figure 12, Figure 13 and Figure 14. Figure 15 and Figure 16 deal with the equivalent stress and total deformation outcomes of the UAV, which was tested under the material property of glass-fibre-reinforced polymer (GFRP)-woven material.

In forward speed operation, the maximum stresses and total deformation occurred at the root of the propellers and landing gear of the UAV.

After the successful completion of forwarding speed, the VTOL operation was computed, wherein the major impacted region of the UAV was higher in VTOL. Therefore, the structural outcome was more crucial than other manoeuvrings, so utmost care was given to this investigation. The CFRP-woven-wet-based composite and GFRP-S-UD-based lightweight material performed better than other lightweight materials. Figure 11 and Figure 12 belong to CFRP’s structural outcomes, and Figure 13 and Figure 14 belong to GFRP’s structural outcomes. As mentioned earlier, two different grid convergence tests were conducted on the induced velocity of the Version I UAV and equivalent stress of the Version II UAV. Figure 13 reflects the comparative analysis of grid convergences test I, wherein the grid convergence conduced on the induced velocity variations while the Version I UAV movement was in the forward direction. From Figure 13, mesh case four is chosen as the best-converged one due to its integrated effects.

The comprehensive structural results of forward speed and VTOL are listed in Table 3 and Table 4.

By comparing the FSI results for HTOL among various materials, FR-4-GFRP-WOVEN has an equivalent stress of 1004.8 MPa and total deformation of 178.31 mm. Since it has the lowest stress and deformation of any other material, FR-4-GFRP-WOVEN has been chosen in the HTOL results. By comparing the FSI results for VTOL among various materials, GFRP-S-UD has an equivalent stress of 15.122 MPa and total deformation of 6.6801 mm. Since it has the lowest stress and deformation of any other material, GFRP-S-UD has been chosen in the VTOL results. Considering both results of VTOL and HTOL, GFRP-S-UD has been chosen as the finalised material for the UAV design because of its high load-withstanding properties.

### 4.3. Discussion as Summary of Observation

As per the research outcomes observed in this work, we have conducted an extensive experimental validation of design, which gives information about various facts considered in the literature as well for conducting experiments. The relations between payload weight, total weight, aspect ratio, and wingspan were derived and the parameters for UAV design were used for the generation of the first BWB model. Some of the initial theoretical calculations are adapted from John Roskam’s modelling [39,40]. CFD simulations are adopted for the first stage of testing, as much of the related literature used the approach predominantly [41]. For the initial design validation, a trial-and-error method was adopted and planned to go through three to four iterations till the design was found to be suitable for the next-generation high-speed use cases of UAVs. Finally, we have presented a noble, innovative idea for designing and developing a blended-wing-body (BWB)-based configuration system for next-generation drone use cases. The proposed method has several benefits, including a very low interference drag, evenly distributed load inside the body, and less radar signature than the other configurations. The main significant characteristic of the BWB airframe is efficient lift and minimal drag, since the BWB airframe has a wide airfoil along with high-lift wings. The next feature is low fuel consumption as well as large payload area. Moreover, the BWB airframe has minimum skin drag since, in this airframe, the total wetted area is reduced. The wing root area is much thicker as compared to conventional design; thereby, optimal weight reduction can be witnessed. The aforementioned experimental result analysis has confirmed the benefits of a BWB-based UAV design for quality-of-service-centric consideration, focusing on next-generation drone use cases.

## 5. Conclusions and Future Scope

In this paper, novel research for designing and developing a blended-wing-body (BWB)-based configuration system for next-generation drone use cases has been presented. The proposed method has several benefits, including a very low interference drag, evenly distributed load inside the body, and less radar signature than the other configurations. Each component design is supported by relevant mathematical derivations as a theoretical proof of concept. The CAD model from the design calculations was generated using CATIA, where the estimated dimensions are predominantly supported. The first iteration of the BWB was conceptually designed with the help of historical relationships and theoretical calculations. Flow analysis over the body and stability analysis was carried out, and based on the results, the second iteration of the model was designed. Flow analysis has been carried out, and results have been generated comparatively with Versions I and II. As an observation, FSI has been carried out, and GFRP-S-UD was found to be the most suitable material as per our analyses. As a limitation, it is also noted that the design needs hardware-based prototype testing with real use-case scenarios. The design parameters’ values might need adjustment considering the comparative analysis of the results from software testing and protype-based hardware testing. In future research, the authors will investigate solutions for these limitations as well as explore the potential real-time use cases of the proposed UAV-assisted framework in smart city environments. Further, the system testing for underwater UAV environments and related design parameter adjustments would also be the quest for future research.

## Figures and Tables

**Table 1:** List of abbreviations.

Abbreviation	Definition
QSPU	Quality-of-Service Provisioning Framework for UAV-assisted networks
UAV	Unmanned Aerial Vehicle
BWB	Blended Wing Body
BWB-UAV	Blended-Wing-Body Unmanned Aerial Vehicle
UDL	UAV Data Link
FSI	Fluid Structure Interaction
CFD	Computational Fluid Dynamics
HTOL	Horizontal Take-off and Landing
VTOL	Vertical Take-off and Landing
CAD Models	Computer-Aided Design Models
CATIA	Computer-Aided Three-Dimensional Application
SIMPLE	Semi-Implicit Method for Pressure Linked Equation
ANSYS	Analysis System
SST	Shear-Stress Transport
NACA	National Advisory Committee for Aeronautics
MH-91	Martin Hepperle 91
UCAV	Unmanned Combat Aerial Vehicle
RANS	Reynolds-Averaged Navier–Stroke
MALE UAV	Medium-Altitude Long-Endurance Unmanned Aerial Vehicle
NLF1015	Natural Laminar Flow (NLF)1015

**Table 2:** Symbol Table.

Symbol	Description
$W_{\mathrm{Pl}}$	Payload weight
$W_{O}$	Overall weight
T	Take-off and landing time
θ	Angle between wings
b	Area of Wing
$C_{L}$	Length of chord
$S_{\mathrm{wing}}$	Wingspan
AR	Aspect ratio
L	Length of fuselage
$L_{\mathrm{UAV}}$	Length of UAV
λ	Taper ratio

**Table 3:** Comparative structural outcomes under forward speed manoeuvring.

Material	Equivalent Stress	Total Deformation
FR-4-GFRP-WOVEN	1004.8 MPa	178.31 mm
CFRP-UD-PREPREG	1311.7 MPa	279.47 mm
Polyethylene	1013.2 MPa	3091.3 mm

**Table 4:** Comparative structural outcomes under VTOL manoeuvring.

Material	Equivalent Stress	Total Deformation
ALUMINIUM ALLOY	15.289 MPa	1.0388 mm
CFRP-UD-PREPREG	30.837 MPa	18.139 mm
CFRP-WOVEN-PREPREG	15.3 MPa	2.119 mm
CFRP-WOVEN-WET	15.283 MPa	2.1923 mm
FR-4-WOVEN-GFRP	34.008 MPa	10.916 mm
GFRP-S-UD	15.122 MPa	6.6801 mm

## Data Availability

Research data will be available on individual requests to the corresponding author considering collaboration possibilities with the researcher or research team and with restrictions that the data will be used only for further research in the related literature progress.
